# Ankle muscle strength discriminates fallers from non-fallers

**DOI:** 10.3389/fnagi.2014.00336

**Published:** 2014-12-19

**Authors:** Thomas Cattagni, Gil Scaglioni, Davy Laroche, Jacques Van Hoecke, Vincent Gremeaux, Alain Martin

**Affiliations:** ^1^Faculté des sciences du sport—UFR STAPS, Université de BourgogneDijon, France; ^2^INSERM unité 1093, Cognition, action et plasticité sensorimotriceDijon, France; ^3^INSERM CIC 1432, Plateforme d’Investigation Technologique, CHU de DijonDijon, France; ^4^Pôle rééducation-réadaptation, CHU de DijonDijon, France

**Keywords:** aging, elderly fallers, postural stability, muscle strength, ankle joint

## Abstract

It is well known that center of pressure (CoP) displacement correlates negatively with the maximal isometric torque (MIT) of ankle muscles. This relationship has never been investigated in elderly fallers (EF). The purpose of this study was thus to analyze the relationship between the MIT of ankle muscles and CoP displacement in upright stance in a sample aged between 18 and 90 years old that included EF. The aim was to identify a threshold of torque below which balance is compromised. The MIT of Plantar flexors (PFs) and dorsal flexors (DFs) and CoP were measured in 90 volunteers: 21 healthy young adults (YA) (age: 24.1 ± 5.0), 12 healthy middle-aged adults (MAA) (age: 50.2 ± 4.5), 27 healthy elderly non-fallers (ENF) (age: 75.5 ± 7.0) and 30 EF (age: 78.8 ± 6.7). The MIT of PF and DF were summed to obtain the overall maximal ankle muscle strength. Body weight and height were used to normalize MIT (nMIT) and CoP (nCoP), respectively. nCoP correlated negatively with nMIT. 90% of EF generated an nMIT <3.1 N·m·kg^−1^, whereas 85% of non-fallers generated an nMIT >3.1 N·m·kg^−1^. The relationship between nMIT and nCoP implies that ankle muscle weakness contributes to increased postural instability and the risk of falling. We observed that below the threshold of 3.1 N·m·kg^−1^, postural stability was dramatically diminished and balance was compromised. Our results suggest that measuring ankle torque could be used in routine clinical practice to identify potential fallers.

## Introduction

Falls are a major concern among older adults. Indeed, approximately 30% of people over 65 years old and 50% of those over 80 years old fall each year (Tinetti et al., 1988; Campbell et al., 1989; O’Loughlin et al., 1993; Hill et al., 1999; Rubenstein and Josephson, 2002). These accidents are a common cause of injuries which can lead to hospitalization and loss of autonomy.

The increase in body sway, generally observed with aging (Sheldon, 1963; Amiridis et al., 2003; Laughton et al., 2003; Onambele et al., 2006; Cavalheiro et al., 2009; Kouzaki and Masani, 2012) has been identified as a major factor responsible for an increased risk of falling among older adults (Fernie et al., 1982; Maki et al., 1994). For instance, Maki et al. (1994) found that, in the year following the measurement of center of pressure (CoP) displacement, elderly people who fell had a significantly greater anteroposterior sway than those who did not fall.

To control body sway while standing upright, humans need to generate appropriate torques at the ankle joint (Horak and Nashner, 1986; Horak et al., 1989; Amiridis et al., 2003, 2005; Loram et al., 2004). During the aging process, it is common to observe a decline in the neuromuscular performance of plantar flexor (PF) and dorsal flexor (DF) muscles (Vandervoort and McComas, 1986; Doherty et al., 1993; Winegard et al., 1996; Simoneau et al., 2007; Billot et al., 2010), which appears to be more pronounced in elderly fallers (EF) than in the rest of the elderly population (de Rekeneire et al., 2003; Perry et al., 2007; LaRoche et al., 2010). The decrease in the maximal strength of ankle muscles may therefore be considered a main cause of postural instability.

Recently, Billot et al. (2010) reported that, in young adults (YA) and elderly non-fallers (ENF) challenged across three postural tasks (i.e., bipedal stance, unipodal stance and tandem stance), a negative linear correlation was observed between the maximal isometric torque (MIT) of ankle muscles and the length of CoP displacement. In the inverted pendulum model, which is generally used to describe upright stance in humans, the equilibrium equation for the foot shows that a variation in muscle torque developed at the ankle joint is directly and linearly translated into a variation of the CoP position (Morasso and Schieppati, 1999). In this model, the fall occurs when the maximal ankle torque capacity of an individual is lower than the torque needed to counterbalance the CoP displacement. Therefore, the analysis of the relationship between CoP displacement and maximal ankle torque in EF could provide valuable insights into balance control in frail elderly subjects. If the relationship between CoP displacement and maximal ankle torque in EF is different from that in non-fallers this would mean that their balance would be even more impaired by the decline in ankle muscle strength than would be expected if the relationship were the same. Should this be the case, it could be supposed that below a certain level of muscle strength, subjects are not able to control their balance. This level of torque could thus be considered an index of the risk of falling.

In view of these considerations, the purpose of this study was to analyze the relationship between MIT of ankle muscles and CoP displacement in a sample aged between 18 and 90 years old that included EF, in order to identify a threshold of torque below which balance is compromised.

## Materials and methods

### Participants

Ninety volunteers, aged between 18 and 90 years old, participated in this experiment. The sample was divided into four groups according to age and history of falls: 21 healthy YA (age: 24.1 ± 5.0), 12 healthy middle-aged adults (MAA) (age: 50.2 ± 4.5), 27 healthy ENF (age: 75.5 ± 7.0) and 30 EF (age: 79.8 ± 6.7). For subjects aged between 60 and 90 years old, the fall history of the previous 6 months was recorded by interview. People who had fallen unexpectedly at least once in the previous 6 months were included in the EF group. The YA and MAA were students or employees at the University of Burgundy. Exclusion criteria for all subjects were muscular disorders, neurological disorders (stroke, multiple sclerosis, Parkinson’s disease), serious visual impairment; body mass index >35, and impaired cognitive status (score of less than 23 on the Mini Mental State Examination). Informed written consent, approved by the local ethics committee, was obtained for all participants after they had been fully informed of all potential risks, discomfort and benefits of the study.

The protocol of the current investigation was approved by the French National Drugs and Health Administration and by the National Ethics Committee section Dijon Est I and was carried out in accordance with the Declaration of Helsinki.

### Data recordings

#### Physical characteristics

Body weight was measured using an electronic scale (SOEHNLE Fitness 7850). Body height was obtained with a measuring rod, with a horizontal slider brought into contact with the vertex while the subject was standing with his back laying against a wall.

#### Maximum voluntary contraction (MVC) torque

Participants were examined in the seated position with the trunk inclined forwards at 40° to the vertical, the knee joint angle at 180° and the ankle joint angle at 90°. Strength was measured with the foot secured by two straps to the footplate of a custom-made ergometer developed by the mechanical workshop of a local engineering school (I.U.T. Génie Mécanique, Dijon, France). The center of rotation of the ergometer shaft was aligned with the anatomical ankle flexion-extension axis. The footplate was connected to a strain-gauge transducer (Société Doerler-Vandoeuvre, France), placed on the axis of the device, and an amplifier (PM Instrumentation, model 1300B, amplification: 8 V = 200 Nm). This apparatus has already been used in previous published studies carried out in our laboratory (Scaglioni et al., 2003; Scaglioni and Martin, 2009). Torque was sampled at a frequency of 2 kHz and processed by a multichannel analog-digital converter (Biopac Systems Inc., USA).

#### Postural stability

Center of pressure displacement during the static postural task was assessed via a force-platform (Stabilotest, TechnoConcept, Cereste, France). This platform was composed of three force sensors which instantaneously measured the coordinates of the point of application of the resultant of the ground reaction forces or CoP. Data from the force-platform were sampled at a frequency of 40 Hz and were synchronized by triggering with the Biopac acquisition system.

### Experimental procedure

All measurements were made in one experimental session lasting approximately 1.5 h. All subjects first carried out the orthostatic postural task and then the MVC task to avoid interference due to fatigue during the postural task.

#### Postural task

During the postural task, subjects stood barefoot on the force-platform for 30 s, with their feet axes forming an angle of 30° (distance between the heels = 2 cm) and with their arms alongside their body. The subjects were asked to remain as still as possible, looking straight ahead at a target point located at eye height and placed at a distance of 150 cm in front of them. This postural task was repeated three times, and a rest period of 1 min was given between trials.

#### Strength task

The MIT for PF and DF was obtained during maximal voluntary isometric contraction tests performed for each leg separately. A 3-min warm-up, which included several submaximal plantar and dorsal flexions, was carried out for each leg. Subjects then performed two maximal contractions lasting 5 s for each leg. A 1-min rest period was systematically given between trials to avoid any fatiguing effect on measurements. If there was a variation of more than 5% between the first and the second MVC, participants were asked to perform an additional MVC. Standardized verbal encouragement was given during attempts to produce the maximal effort.

### Data analysis

#### MVC torque

Only the highest PF and DF MIT were considered for analysis. For each contraction type, the torques achieved by both legs were summed to obtain the overall force production capacity at the ankle joint. The MIT was then normalized for the body weight (nMIT).

#### CoP displacement

During the postural task, the 95% elliptical area (EA) containing 95% of the CoP sampled positions, the antero-posterior (YCoP), the mediolateral (XCoP) and the total length of CoP displacement (TCoP) were recorded for 30 s. These parameters were expressed as an average of three measurements. Each parameter of the CoP displacement was normalized for the height (nEA, nYCoP, nXCoP and nTCoP).

### Statistical analysis

All statistical tests were performed with SPSS software (SPSS, Inc., Chicago, IL, USA). Data are presented as means ± standard deviations (SD). The critical level for statistical significance was set at 5%. A multivariate analysis of variance (MANOVA) was performed in all outcome variables with group (YA, MAA, ENF and EF) as factor. When a main effect was found, Tukey’s *post hoc* analysis was performed. The best degree of association between PF + DF nMIT and nTCoP was determined by applying a linear regression analysis and a log-linear correlation test. Receiver operating characteristic (ROC) curves were used for the assessment of sensitivity and specificity of PF + DF nMIT to predict the risk of falling. The area under the curve and the optimal cut-off value were calculated. The area under the curve describes the test’s overall performance. A value of 1 indicates perfect discrimination, whereas a value of 0.5 indicates poor discrimination; a value >0.7 is considered satisfactory discrimination. We chose the optimal cut-off by using the *Youden* *index*. The Youden Index, calculated as follows: sensitivity + (specificity −1), is ranging from 1 to 0, with 1 representing perfect accuracy and 0 the accuracy that would asymptotically be achieved through chance. This index, a common summary measurement of the ROC curve, represents the maximum potential effectiveness of a marker. The value providing a sensitivity of 1 (i.e., value for which all subjects are EF) and the value giving a specificity of 1 (i.e., the value below which all subjects are non-fallers) were also estimated.

## Results

### Subjects’ descriptive characteristics

The main characteristics of participants are described in Table 1. There was no statistical difference between the age of EF and ENF. Height was lower in both senior groups than in YA and MAA groups (*P* < 0.01). Body weight was higher in MAA than in the other groups (*P* < 0.05). Despite differences between height and weight, BMI was equivalent across the four groups.

**Table 1:** **Subjects’ characteristics and strength and balance performances**.

	Young adults range = 18–34 years	Middle-aged adults range = 44–59 years	Elderly non-fallers range = 62–87 years	Elderly fallers range = 64–89 years
Age, year	24.1 ± 5.0	50.2 ± 4.5^*^	75.5 ± 7.0^*$^	79.8 ± 6.7^*$^
Body weight, kg	72.5 ± 7.8	74.8 ± 16.3	63.6 ± 11.6^$^	63.7 ± 12.4^$^
Height, cm	180.4 ± 5.9	174.7 ± 8.8	164.0 ± 7.7^*$^	160.4 ± 8.6^*$^
Body mass index (kg/m^2^)	22.3 ± 2.2	24.6 ± 5.4	23.5 ± 3.4	24.7 ± 4.1
PF MIT, N·m	320.4 ± 64.7	235.4 ± 65.8*	164.6 ± 63.2^*$^	105.5 ± 34.8^*$†^
PF nMIT, N·m·kg^−1^	4.4 ± 0.7	3.2 ± 0.7*	2.5 ± 0.7^*$^	1.7 ± 0.5^*$†^
DF MIT, N·m	89.6 ± 13.8	75.9 ± 20.2	57.7 ± 19.2	42.7 ± 9.9^*^
DF nMIT, N·m·kg^−1^	1.2 ± 0.1	1.0 ± 0.2	0.9 ± 0.2	0.7 ± 0.2^*^
PF + DF MIT, N·m	410.0 ± 74.3	311.3 ± 72.7*	222.2 ± 78.2^*$^	148.2 ± 40.3^*$†^
PF + DF nMIT, N·m·kg^−1^	5.6 ± 0.8	4.2 ± 0.7*	3.4 ± 0.8^*$^	2.4 ± 0.6^*$†^
TCoP displacement, mm	297.9 ± 80.8	344.3 ± 89.5	410.7 ± 137.5	744.0 ± 405.6^*$†^
nTCoP	1.7 ± 0.4	2.0 ± 0.5	2.5 ± 0.8	4.7 ± 2.4^*$†^
XCoP displacement, mm	167.0 ± 56.9	159.7 ± 34.8	227.2 ± 103.7	309.8 ± 152.3^*$†^
nXCoP	0.9 ± 0.3	0.9 ± 0.2	1.4 ± 0.6	2.0 ± 0.9^*$†^
YCoP displacement, mm	208.8 ± 61.0	271.5 ± 78.3	290.4 ± 91.5	576.5 ± 324.5^*$†^
nYCoP	1.2 ± 0.3	1.6 ± 0.5	1.8 ± 0.5	3.6 ± 1.9^*$†^
EA, mm^2^	113.0 ± 50.8	123.4 ± 76.3	185.1 ± 100.9	279.6 ± 152.5^*$†^
nEA (EA/size), mm	0.6 ± 0.3	0.7 ± 0.4	1.1 ± 0.6	1.7 ± 0.9^*$†^

MIT: maximal isometric torque; nMIT : maximal isometric torque normalized for weight; PF: Plantar flexion; DF: Dorsal flexion; TCoP: center of pressure displacement; nTCoP: center of pressure displacement normalized for height; XCoP: Mediolateral center of pressure displacement; nXCoP: Mediolateral center of pressure displacement normalized for height; YCoP: Anteroposterior center of pressure displacement; nYCoP: Anteroposterior center of pressure displacement normalized for height; EA: 95% Ellipse area of the center of pressure displacement; nEA: 95% Ellipse area of the center of pressure displacement normalized for height. Value are means ± SD. *Significant difference from young subjects, *P* < 0.05. ^$^ Significant difference from middle-aged group, *P* < 0.05. ^†^ Significant difference from elderly non-fallers, *P* < 0.05.

### Maximal isometric torque

As shown in Table 1, PF MIT and nMIT were lower in EF than in the other groups (*P* < 0.001). When considering the three non-faller groups, we observed that age gradually affected PF MIT (i.e., YA > MMA, *P* < 0.001; MMA > ENF, *P* < 0.001) and nMIT (i.e., YA > MMA, *P* < 0.001; MMA > ENF, *P* < 0.05). In dorsiflexion, MIT and nMIT were lower in EF than in YA (*P* < 0.001), while no difference was observed across the three non-faller groups and for MAA, ENF and EF.

The effect of age was more marked when the sum of PF and DF was considered. Indeed, among the three non-faller groups, age gradually affected PF + DF MIT (i.e., YA > MMA, *P* < 0.001; MMA > ENF, *P* < 0.001) and PF + DF nMIT (i.e., YA > MMA, *P* < 0.001; MMA > ENF, *P* < 0.05). Finally EF were weaker than YA, MAA and ENF (*P* < 0.001).

### CoP displacement

As shown in Table 1, when considering only the three non-faller groups (i.e., YA, MAA, ENF) none of the variables of CoP displacement (i.e., EA, YCoP, XCoP, TCoP) or CoP displacement normalized for height (nEA, nYCoP, nXCoP, nTCoP,) were affected by age. However, all of the CoP parameters were significantly higher in EF than in the other groups (YA < EF, *P* < 0.001; MAA < EF, *P* < 0.001; ENF < EF, *P* < 0.001).

### Correlation analysis

**Figure 1:**
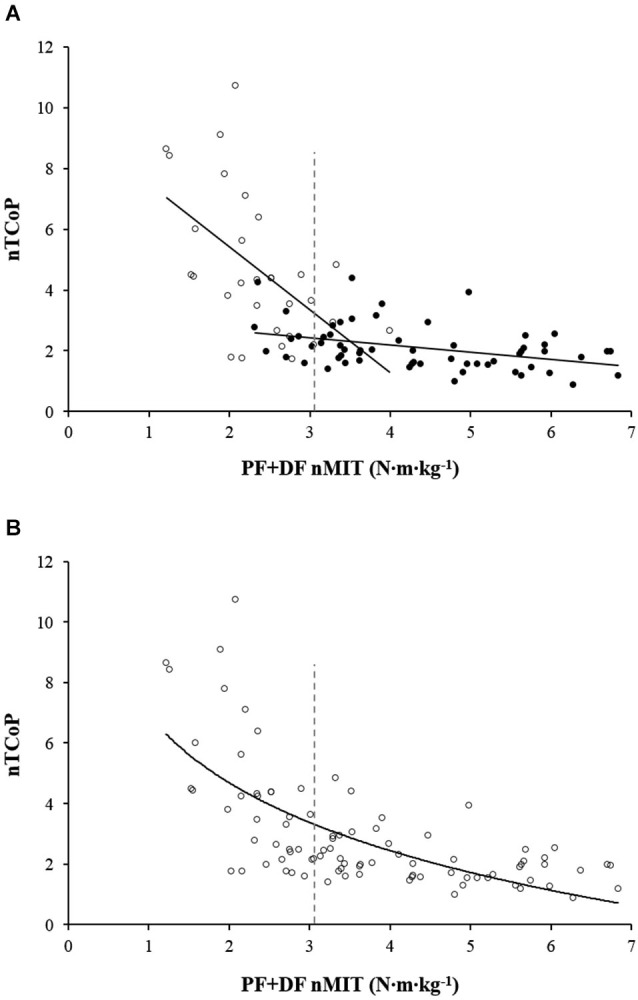
**Relationship between TCoP displacement normalized for height (nTCoP) and the sum of plantar (PF) and dorsal (DF) flexors maximal isometric torque normalized for body weight (FP + FD nMIT)**. **(A)** represents the linear relationship between nTCoP and PF + DF nMIT for non-fallers (filled circle) and elderly fallers (EF) (empty circle). The linear regressions equations are *nTCoP = −0.24·(FP + FD nMIT)+3.14* (*r* = 0.40, *P* < 0.001) for non-fallers and *nTCoP = −2.07·(FP + FD nMIT) + 9.56* (*r* = 0.55, *P* < 0.001) for EF. The vertical gray dashed line represents the cut-off value of PF + DF nMIT that distinguishes between EF and non-fallers. It corresponds to the highest *Youden index* obtained from receiver operating characteristic analysis. **(B)** represents the logarithmic relationship between nTCoP and PF + DF nMIT for the whole sample; the equation is: *nTCoP = −7.44·log10(FP + FD nMIT) + 6.93*, (*r* = 0.68, *P* < 0.001). The vertical gray dashed line represents the cut-off value of PF + DF nMIT that distinguishes between EF and non-fallers. It corresponds to the highest *Youden index* obtained from receiver operating characteristic analysis.

As depicted in Figure 1A, when data for EF were analyzed separately from the rest of the sample, the two relationships were well described by linear regressions (Non-fallers: nTCoP = −0.24·(FP + FD nMIT) + 3.14, *r* = 0.40, *P* < 0.01; EF: nTCoP = −2.07·(FP + FD nMIT) + 9.56, *r* = 0.55, *P* < 0.01). The regression lines for fallers and non-fallers were found to intersect and the x-coordinate of the interception point was 3.4 N·m·kg^−1^. When the whole sample was considered, the function that best described the relationship between nTCoP and FP + FD nMIT was curvilinear (linear: nTCoP = −0.81·(FP + FD nMIT) + 5.95, *r* = 0.60, *P* < 0.001; curvilinear: nTCoP = −7.44·log10(FP + FD nMIT) + 6.93, *r* = 0.68, *P* < 0.001; Figure 1B).

### ROC analysis

**Figure 2:**
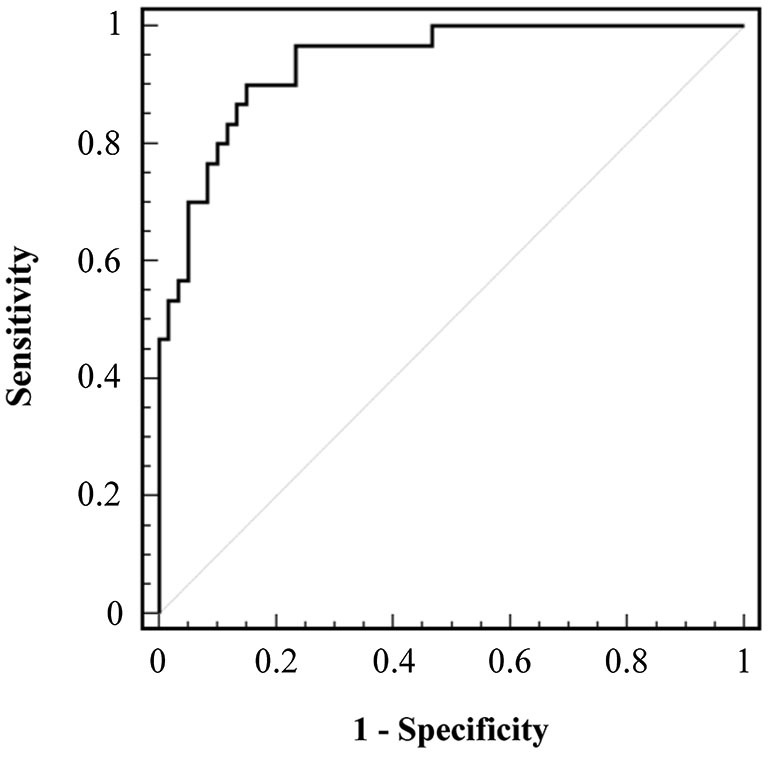
**Receiver Operating Characteristic (ROC) curve of the sum of maximal isometric torque of plantar flexion and dorsal flexion normalized for body weight (PF + DF nMIT)**. ROC curve of the sensitivity against the false-positive rate (1—specificity) plotted across a range of thresholds is represented. The major diagonal represents an area of curves equal to 0.5 and a prediction no better than chance. The black line represents the results taken from the PF + DF nMIT measurements.

Figure 2 shows the ROCs analysis carried out for PF + DF nMIT values of the whole sample. The area under the ROC curve was 0.94, indicating that FP + FD nMIT is an appropriate measurement to discriminate EF from non-fallers. The highest *Youden index* (0.75) was observed at a value of 3.1 N·m·kg^−1^, which corresponded to a sensitivity of 90% and a specificity of 85%. In other words, the maximal muscle strength for 90% of EF was <3.1 N·m·kg^−1^, while it was >3.1 N·m·kg^−1^ for 85% of non-fallers (YA, MAA and ENF). In addition, a sensitivity of 1 was obtained for a value >4.0 N·m·kg^−1^, meaning that subjects able to generate a torque >4.0 N·m·kg^−1^ were all non-fallers. A specificity of 1 was obtained for a value <2.2 N·m·kg^−1^, indicating that all subjects able to generate a maximum torque <2.2 N·m·kg^−1^ were fallers.

## Discussion

The purpose of this study was to analyze the relationship between MIT of ankle muscles and CoP displacement in a sample aged between 18 and 90 years old that included EF. The aim was to identify a torque threshold that discriminated between fallers and non-fallers. We found a negative curvilinear correlation between these parameters, which suggests that the decrease in ankle muscle strength is one of the factors responsible for impaired postural stability. In addition, our analysis indicated that a value of 3.1 N·m·kg^−1^ discriminated between fallers and non-fallers with a *Youden index* of 0.75. This torque value represents a threshold below which all patients were fallers and body sway increased dramatically.

### MVC torque

Because statistical analysis showed similar changes in MIT and nMIT, to simplify matters in this paragraph we only used the abbreviation MIT. The present results showed that age gradually affected PF + DF MIT, thus corroborating previous findings (Vandervoort and McComas, 1986). Moreover, PF + DF MIT was far lower in the elderly participants with a history of falls than in YA (−58%), MAA (−44%) and ENF (−31%). However, by analyzing the PF MIT and DF MIT separately, the statistical analysis showed that these muscle groups were affected differently by aging. Indeed, when the EF group was excluded from the statistical analysis, the present study corroborated what was observed in earlier investigations, namely a decrease in PF MIT with aging (Winegard et al., 1996; Simoneau et al., 2005, 2007, 2009; Billot et al., 2010) and good preservation of DF MIT (Winegard et al., 1996; Lanza et al., 2003; Simoneau et al., 2009; Billot et al., 2010). The preservation of DF MIT has been explained by referring to the age-related decrease in triceps surae coactivation during maximal dorsiflexion (Simoneau et al., 2009). Indeed, these authors showed that when the mechanical contribution of antagonist muscles (i.e., PFs) is removed from the resultant MIT, the performance of DF is altered by aging in the same way as that of PF. In contrast, when we compared the results of EF with ENF, we found a decrease of 34% and 24% for PF and DF MIT, respectively. Our results are in keeping with those obtained by LaRoche et al. (2010), who found that the PF MIT and DF MIT of older female fallers was 27% and 16% lower, respectively, than that in older female non-fallers. Some authors found selective impairment of DF (Whipple et al., 1987; Skelton et al., 2002) or PF (Sieri and Beretta, 2004; Perry et al., 2007) in EF, while others did not report any difference between the two samples (Melzer et al., 2004; Bento et al., 2010; Crozara et al., 2013). It is likely that these discrepancies may be ascribed to differences in the sample characteristics (e.g., disparity of age, physical traits: weight, height, body mass index) or to the method used to evaluate the maximal torque production capacity (isometric vs. dynamic).

### CoP displacement

Because statistical analysis showed similar changes in CoP and normalize CoP (nCoP), to simplify matters in this paragraph we only used the abbreviation CoP. CoP displacement, whatever the parameter used to quantify it (i.e., EA, YCoP, XCoP, TCoP), was statistically similar among the three age groups of non-fallers (YA, MAA and ENF). Although this observation is in keeping with the results of some earlier studies (Shumway-Cook et al., 1997; Simoneau et al., 2008; Billot et al., 2010), several authors found an increase in CoP displacement with aging (Amiridis et al., 2003; Laughton et al., 2003; Onambele et al., 2006; Cavalheiro et al., 2009; Kouzaki and Masani, 2012). It should be pointed out that the lack of a significant difference between YA and ENF shown by our analysis could be ascribed to inter-subject variability, which was greater in ENF (coefficient of variation for TCoP displacement: 33.5%) than in YA (coefficient of variation for TCoP displacement: 27.1%).

Furthermore, our results showed that in EF, CoP displacement was greater than that in ENF, whatever the parameter considered (i.e., EA, YCoP, XCoP, TCoP). This finding confirms the general observation of the literature, that is to say greater postural sway in EF than in ENF (Fernie et al., 1982; Maki et al., 1994; Muir et al., 2013). However, also in this case, some differences have emerged. Indeed, among studies that distinguished between YCoP and XCoP, some showed a greater displacement for EF only in the anteroposterior direction (Maki et al., 1994; Pajala et al., 2008), while others reported greater body sway in the frontal plane (Bergland et al., 2003; Bergland and Wyller, 2004). Despite these discrepancies, the general finding from the literature and our results is that elderly people, and especially those with a history of falls, have greater postural sway than their younger counterparts.

### Relationship between nTCoP and PF + DF nMIT

The finding that elderly subjects have weaker ankle muscles than younger subjects, and that this weakness is more pronounced in EF, suggests that a deficit in strength may cause an increase in postural sway and consequently compromise the ability to maintain postural stability. Our results showed that, whatever the group of subjects (YA, MAA, ENF and EF), there was a significant negative correlation between nMIT of ankle muscles and nTCoP displacement. This confirms the findings of Kouzaki and Masani (2012), who found a correlation between soleus volume and the mean velocity of TCoP displacement. This obviously implies that the weakest individuals are those who oscillate most. Furthermore, Billot et al. (2010) showed that the more complex the postural task, the greater the TCoP displacement and the stronger the correlation between MIT and TCoP. This implies that the greater the instability (i.e., the more complex the postural task), the greater the dependence of balance on muscle strength.

More specifically, the present analysis showed that, for the sample taken as a whole, the comprehensive relationship between nMIT and nTCoP is curvilinear. In contrast, if EF are separated from non-fallers (i.e., YA, MAA and ENF), the data are better fitted by two linear regression lines. The regression line for EF presented a higher correlation coefficient and a steeper slope than that for non-fallers. This suggests that diminished muscle strength has a greater functional impact in EF than in non-fallers because it induces a greater increase in nTCoP displacement. The present result supports our initial hypothesis of a slope failure in the general nMIT-nTCoP relationship provoked by the addition of data from EF which again leads us to the conclusion that the greater the instability of an individual (i.e., elderly faller) the more his balance depends on ankle muscle strength. Previous studies showed that the age-related changes in postural control induce an increased level of muscle activity across lower-limb joints in older adults (Laughton et al., 2003; Simoneau et al., 2008). More recently, Billot et al. (2010) observed a positive linear relationship between TCoP displacement and the relative PF + DF torque (i.e., % PF + DF MIT) needed to maintain the upright stance. In other words, individuals who oscillate more need greater strength, relative to their maximal capabilities, to preserve their balance during the upright posture and this is critical especially for EF, whose maximal capabilities are particularly impaired. The greater muscle involvement may increase energy expenditure to maintain the upright posture. This could induce premature fatigue, which in turn could amplify body sway (Boyas et al., 2013) and as a result increase the risk of falling.

### Critical torque values

We used the ROC analysis to identify a critical MIT value to discriminate between fallers and non-fallers, that is to say a threshold below which, the risk of falling is markedly increased. The analysis revealed that subjects who did not report any previous falls had a PF + DF nMIT >4.0 N·m·kg^−1^. For the clinician, this value could represent a first alert threshold, indicating the necessity to be particularly attentive to the progressive muscle weakening of patients who fall below this level of strength.

The highest Youden index was found for a 3.1 N·m·kg^−1^ value, corresponding to a sensitivity of 90% and a specificity of 85%, meaning that 90% of the subjects with a PF + DF nMIT under 3.1 N·m·kg^−1^ were fallers and 85% of subjects with a PF + DF nMIT above this value were non-fallers. The value of 3.1 N·m·kg^−1^ was close to the intersection point of the regression lines found for fallers and non-fallers (*x*-coordinate: 3.4 N·m·kg^−1^), thus confirming the accuracy of this index to detect potential fallers. This point could be considered a second alert threshold, indicating a high risk of falling for patients and the need to improve muscle strength. Finally, the ROC analysis showed that subjects with a PF + DF nMIT <2.2 N·m·kg^−1^ were all EF. For these patients, strengthening the ankle muscles is an absolute priority. It has been widely demonstrated that strength training programs that focus on postural muscles may improve balance (Topp et al., 1993; Amiridis et al., 2005; Orr et al., 2008; Gonzalez et al., 2014). In particular, Amiridis et al. (2005), who focused strength training specifically on an ankle muscle (i.e., tibialis anterior), found that a 4-week conditioning period improved DF MIT and decreased postural sway. These previous findings and our results highlight the importance of setting-up strength training programs based on ankle muscles to improve postural stability and prevent the risk of falling in older people.

In conclusion, the current investigation demonstrated that the maximal torque of ankle muscles is an easy and effective indicator of the risk of falling. A particularly interesting aspect that emerges from the present analysis is the threshold of alert that corresponds to a critical strength value (i.e., 3.1–3.4 N·m·kg^−1^), below which postural stability is severely impaired. The identification of a threshold that makes it possible to discriminate between fallers and non-fallers clearly indicates that weakness of the ankle plantar flexor and dorsal flexor muscles markedly aggravates postural instability. Including the assessment of ankle muscle strength in routine clinical practice therefore seems to be crucial so as to detect the risk of falling, which is an extremely disabling event for older adults. Finally, following the diagnosis of muscular weakness, patients should be steered towards an appropriate fall prevention program specifically designed to enhance the strength of postural muscles.

## Conflict of interest statement

The authors declare that the research was conducted in the absence of any commercial or financial relationships that could be construed as a potential conflict of interest.
